# β-Glucans, *Pneumocystis jirovecii* and Atherogenic Inflammation: From Pulmonary Immunity to Cardiovascular Risk

**DOI:** 10.3390/jof12060434

**Published:** 2026-06-14

**Authors:** José C. Castillo, Enrique Iglesias, Johanna Castillo, Luis Fonte, Carlos E. Aragón-López, Claudia L. Cueto-Aragón, Jaime Palomares-Marín, Gabriela G. Carrillo-Núñez, Bryan Ortiz, Luis M. Beltrán-Romero, Héctor R. Pérez-Gómez, Yaxsier de Armas, Enrique J. Calderón

**Affiliations:** 1 Division of Health Sciences, Graduate School, Keiser University, West Palm Beach, FL 33409, USA; josecastillonunez@student.keiseruniversity.edu; 2 Centro de Ingeniería Genética y Biotecnología, Havana 11400, Cuba; enrique.iglesias@cigb.edu.cu; 3 Clínica Médica de Zamora, Cancún 77533, Mexico; dra.johannacn@gmail.com; 4 Centro de Investigación, Diagnóstico y Referencia, Instituto de Medicina Tropical “Pedro Kourí”, La Habana 11400, Cuba; luisfonte@infomed.sld.cu; 5 Departamento de Ciencias Agronómicas y Veterinarias, Instituto Tecnológico de Sonora, Unidad Náinari, Sonora 85137, Mexico; carlos.aragon139216@potros.itson.edu.mx; 6 Hospital Infantil “Eva Sámano de López Mateos”, Morelia 58253, Mexico; claudiacueto22@gmail.com; 7 Departamento de Microbiología y Patología, Centro Universitario de Ciencias de la Salud, Universidad de Guadalajara, Guadalajara 44100, Mexico; jaime.palomares@academicos.udg.mx (J.P.-M.); gabriela.carillo@cucs.udg.mx (G.G.C.-N.); 8 Instituto de Investigaciones en Microbiología, Facultad de Ciencias, Universidad Nacional Autónoma de Honduras, Tegucigalpa 11101, Honduras; bryan.ortiz@unah.edu.hn; 9 Instituto de Biomedicina de Sevilla, Hospital Universitario Virgen del Rocío/Consejo Superior de Investigaciones Científicas/Universidad de Sevilla, 41013 Sevilla, Spain; lbeltran@us.es; 10 Instituto de Patología Infecciosa y Experimental “Francisco Ruiz Sánchez”, Centro Universitario de Ciencias de la Salud, Universidad de Guadalajara, Guadalajara 44100, Mexico; hrulito@hotmail.com; 11 Departamento de Anatomía Patológica, Instituto de Medicina Tropical “Pedro Kourí”, La Habana 11400, Cuba; 12 Centro de Investigación Biomédica en Red de Epidemiología y Salud Pública (CIBERESP), 28029 Madrid, Spain

**Keywords:** *Pneumocystis jirovecii*, atherogenic inflammation, β-D-glucans, cardiovascular risk

## Abstract

The interaction between *Pneumocystis jirovecii* and systemic inflammation has emerged as a potential modulator of cardiovascular risk. This review describes the potential of β-glucans to contribute to atherogenic inflammation. A narrative review was developed on the PubMed/MEDLINE, Scopus, Web of Science and Google Scholar databases. The inflammatory pathways induced by β-glucans from *P. jirovecii* contrast with the immunometabolic effects of dietary β-glucans. The relevance of serum (1→3)-β-D-glucans as a marker of systemic exposure was also described, although it is not specific to *P. jirovecii*. *P. jirovecii* β-glucans activate Syk–CARD9–NFκB, MAPK and STAT3 signalling pathways. This signalling promotes proinflammatory monocyte/macrophage polarization and a systemic microenvironment of low-grade inflammation with proatherogenic potential. The serum persistence of (1→3)-β-D-glucan indicates prolonged exposure, even in the absence of overt clinical manifestations of colonization. Conversely, dietary β-glucans have been observed to elicit regulatory effects facilitated by microbiota and metabolism. In experimental setting, a causal link has been established between fungal β-glucans and atherosclerosis. *P. jirovecii* β-glucans act as immunological mediators capable of amplifying pulmonary and systemic inflammation, constituting a possible modulator of cardiovascular risk. Distinguishing between fungal and dietary β-glucans is imperative for comprehending emerging mechanisms of vascular inflammation.

## 1. Introduction

Systemic inflammation plays a pivotal role in understanding cardiovascular vulnerability and the progression of atherosclerosis, a chronic condition with a substantial global burden [1]. In this context, *Pneumocystis jirovecii* should be considered a relevant factor due to the immunomodulatory properties of its β-glucans, structural components capable of activating pattern recognition receptors, such as Dectin-1 and EphA2, and inducing proinflammatory signaling cascades [2,3]. In general, fungal β-glucans are recognized as modulators of innate and adaptive immunity. These modulators have the ability to promote cytokine secretion and the activation of multiple immune pathways [4]. It has been demonstrated that persistent exposure to these polysaccharides, even in scenarios of subclinical colonization—as described in populations with chronic respiratory diseases or immunological vulnerability—could contribute to the sustained modulation of local inflammatory pathways and the configuration of an altered immune microenvironment [5]. This link is relevant given the high prevalence of atherosclerosis and growing evidence that colonization by *P. jirovecii* is more prevalent than is clinically recognized, with varying prevalences reported in different vulnerable populations and in chronic respiratory diseases [5,6,7].

Despite the mounting interest in the immunomodulatory role—predominantly immunostimulatory—of *P. jirovecii* β-glucans, there are currently no epidemiological studies directly evaluating the incidence of major cardiovascular events in populations with a high prevalence of infection or colonization by this fungus. The extant evidence derives primarily from experimental and observational studies that describe the activation of inflammatory pathways induced by β-glucans and the biological changes associated with colonization in chronic respiratory diseases. These findings suggest a possible relationship that warrants further evaluation using population cohorts and prospective designs [1,2,3,4,5,6,7].

In order to contextualize these possible links, it is pertinent to review the biological and immunological characteristics of *P. jirovecii*. This human-specific opportunistic fungus exhibits a clinical spectrum that ranges from subclinical states to overt respiratory symptoms [8,9]. The life cycle of this organism comprises two morphotypes that exhibit divergent immunological manifestations. Cysts, the infective form, are characterized by a cell wall that is rich in branched β-glucans (β-1,3 and β-1,6). In contrast, trophic forms are devoid of these polysaccharides [10,11]. This distinction is salient because the β-glucans present in cysts, in contrast to the predominant surface glycoprotein, which does not stimulate dendritic cells, serve as the primary catalyst for immune recognition and the initiation of inflammatory pathways [10]. Trophic forms induce adaptive responses with minimal inflammatory activation. This promotes their persistence in subclinical settings [11]. Overall, the release of β-glucans from cysts during active infection represents a key mechanism of immune activation, associated with local inflammation and, in contexts of established disease, potentially linked to systemic manifestations [8,9].

These processes are of particular interest within the context of atherosclerosis, a chronic inflammatory disease characterized by the accumulation of lipids and immune cells within the arterial wall. The sustained activation of monocytes, macrophages, and endothelial cells constitutes a pivotal element in the initiation and progression of atherosclerotic lesions, as it promotes the amplification of local inflammatory responses [12]. In this context, low-grade systemic inflammation—as reflected in inflammatory indices derived from circulating cell populations—is recognized as a pathophysiological link between distal immune stimuli and vascular alterations, including endothelial dysfunction [13,14].

The intricate interplay between *P. jirovecii* β-glucans and inflammatory processes, coupled with the methodological heterogeneity of the extant evidence, warrants a narrative approach that aims to integrate pathophysiological, experimental, and clinical dimensions [12]. This review examines, in an integrative—though not exhaustive—manner, how these β-glucans, characterized by a predominantly immunostimulatory profile, could directly or indirectly contribute to atherosclerotic processes in vulnerable populations [10,11,12]. The analysis focuses on immunological and vascular mechanisms, without addressing therapeutic interventions or making quantitative comparisons. In this context, the proposed synthesis aims to provide a conceptual framework for interpreting plausible mechanisms, contextualizing emerging findings, and guiding both the interpretation of biomarkers and the design of future research in fungal immunology and vascular biology [12,14]. It is imperative to underscore that extant evidence is predominantly derived from experimental studies, preclinical models, and indirect clinical observations; consequently, the relationships discussed herein should be interpreted as biologically plausible associations rather than clinically established causal links [12,13,14].

## 2. Materials and Methods

A narrative review was conducted to integrate representative evidence on the potential associations between *Pneumocystis jirovecii* β-glucans and inflammatory processes implicated in atherogenesis. This review considered their interaction with pattern recognition receptors, including Dectin-1, various lectin-like receptors (CLRs), and EphA2, in macrophages, monocytes, and lung epithelium. This framework was developed to address the conceptual and exploratory nature of the objectives. It was intentionally non-exhaustive aligning with the recommendations of the International Committee of Medical Journal Editors (ICMJE) for narrative reviews with an integrative purpose [15].

The design of the review followed the principles of synthesis described by Baumeister and Leary [16], with the objective of conceptual construction and the development of explanatory frameworks in the absence of homogeneous evidence. In addition, elements of the methodological proposal by Green, Johnson, and Adams [17] were incorporated, emphasizing the clear delimitation of the topic, the explicit definition of the scope, and the progressive integration between literature review and analytical writing.

In accordance with these guidelines, the review team methodically focused the analysis on immunological, pulmonary, and vascular mechanisms associated with atherogenic inflammation, with the objective of preventing thematic dispersion and maintaining alignment with the central question of the manuscript.

A comprehensive literature search was conducted in PubMed/MEDLINE, Scopus, Web of Science, and Google Scholar, using combinations of terms related to *P. jirovecii*, β-glucans (β 1,3 and β 1,6) and other cyst wall polysaccharides, pulmonary immunity, systemic inflammation, atherosclerosis, pattern recognition receptors (Dectin-1, CLRs, EphA2), microbiota, metabolism, and serum (1→3)-β D glucan. The following types of documents were included: original articles, specialized reviews, experimental studies, relevant clinical reports, and consensus documents published in English or Spanish.

The selection of sources was based on their conceptual relevance, methodological rigor, scientific validity, and contribution to the proposed pathophysiological framework. Although no explicit time limits were stipulated, priority was accorded to recent publications that reflected contemporary advances in the domains of fungal immunology, vascular biology, metabolism, microbiota, and atherogenic inflammation.

Information was extracted, compared, and organized through an iterative process that allowed for the refinement of findings and the evaluation of convergences and discrepancies between studies. During this process, the evidence was structured around three main analytical themes:

1. The role of *P. jirovecii* β-glucans as triggers of pulmonary and systemic inflammation.
2. The modulatory or cardioprotective effects of dietary or fungal β-glucans on microbiota, metabolism, inflammation, and atherosclerosis must be considered.
3. The presence and relevance of serum (1→3)-β D glucan as a marker of systemic exposure in the context of *P. jirovecii* should be acknowledged. Importantly, other types of glucans exist; however, this biomarker is the available and standardized tool for assessing systemic fungal exposure.

These three axes functioned as a conceptual framework for integrating the different levels of evidence and exploring possible pathophysiological relationships between antifungal immunity, vascular inflammation, and cardiovascular risk.

In accordance with this methodological approach, the manuscript was organized in stages. First, *P. jirovecii* is biologically/clinically important as a pathogen beyond β-glucans. Also, the structural and immunological characteristics of *P. jirovecii* β-glucans are analyzed and then contrasted with dietary β-glucans. In the following section, the primary inflammatory pathways that are activated by these polysaccharides in macrophages and lung epithelium will be delineated. Additionally, their potential contribution to a proatherogenic phenotype will be discussed. On this basis, the clinical implications related to the persistence of circulating β-glucans and their potential systemic impact are explored, and finally, experimental models that could allow the evaluation of causal relationships between exposure to *P. jirovecii* β-glucans and atherogenic inflammation are discussed.

Conflicts of interest were declared and managed following the guidelines of the World Association of Medical Editors (WAME), ensuring transparency and adherence to international ethical standards [18]. No external funding was received. Literature selection relied solely on academic search engines.

## 3. Results and Discussion

### 3.1. P. jirovecii Is Biologically/Clinically Important as a Pathogen Beyond β-Glucans

*Pneumocystis jirovecii* is an atypical, extracellular, non-cultivable fungus that has traditionally been associated with pneumonia in immunocompromised individuals, named *Pneumocystis* pneumonia (PCP). Despite existing antiretroviral therapy and chemoprophylaxis, *P. jirovecii* remains one of the most significant pathogens in HIV patients. Due to its impact on health—its global annual incidence stands at 505,000 cases, with an associated mortality rate of 42.4—*P. jirovecii* has been included in the World Health Organization’s Fungal Priority Pathogens List, within the medium-priority group [19].

Colonization by *Pneumocystis jirovecii*, defined as the presence of the fungus in the respiratory tract at low levels without causing symptoms of active pneumonia, has a significant clinical and epidemiological impact on both healthy patients and those with pre-existing conditions. Although historically considered a harmless commensal in healthy individuals, medical evidence shows that colonization acts as a driver of inflammation, a factor in the progression of chronic diseases, and a critical source of pulmonary transmission. Reported prevalence rates of colonization range from 4.3% in cancer patients to 55% in chronic obstructive pulmonary disease [19].

### 3.2. The Structural and Immunological Characteristics of Pneumocystis jirovecii β-Glucans Are Examined and Contrasted with Those of Dietary β-Glucans

**Figure 1:**
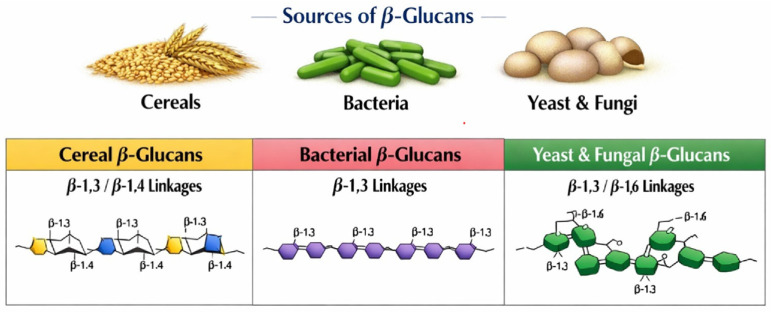
Structural diversity of β glucans from distinct biological sources. The figure presents representative chemical structures of β glucans derived from cereals, bacteria, and yeast/fungi. Cereal β glucans are depicted as linear polymers composed of glucose units linked through β-(1 3) and β-(1 4) glycosidic bonds. Bacterial β glucans are illustrated as unbranched chains containing exclusively β-(1 3) linkages. Yeast and fungal β glucans are characterized by a branched configuration, consisting of a β-(1 3) backbone with side chains connected via β-(1 6) bonds. These structural distinctions are of biological relevance, as they influence physicochemical properties such as solubility and conformational stability, as well as functional outcomes including immunomodulatory activity and metabolic regulation.

Beta-glucans are non-starch polysaccharides found in the cell walls of fungi, plants (oats, barley), yeast, and bacteria. Their structural heterogeneity (Figure 1) directly dictates their solubility, binding affinity, and subsequent biological activities [20]. These glucose polymers differ in structure depending on their origin (Figure 1), with cereal β-glucans containing β-(1,3) and β-(1,4) linkages, while fungal β-glucans have β-(1,3) and β-(1,6) bonds [20].

**Figure 2:**
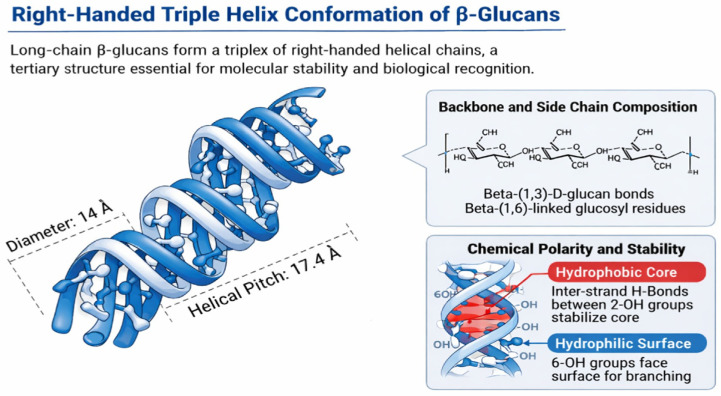
Triple helix conformation of β glucans. The diagram illustrates the right-handed triple helix formed by β glucans, composed of three intertwined β 1→3 D glucan chains with β 1→6 linked glucosyl side residues. The helical structure has a diameter of approximately 14 Å and a pitch of 17.4 Å. Stability is conferred by interstrand hydrogen bonds involving 2 OH groups, which generate a hydrophobic core, while 6 OH groups project outward to form a hydrophilic surface that accommodates branching. This molecular architecture underlies the physicochemical properties and biological recognition of β glucans, including their immunomodulatory activity.

The frequency and length of side chains vary wildly. Yeast-derived glucans feature complex, frequent branching, while fungal glucans (like lentinan) possess specific branching ratios (1 branch per 3 backbone residues). Polymetric chains range from low molecular weight oligosaccharides (a few kilodaltons) to high molecular weight polymers exceeding 10^6^ Daltons. Finally, in solution, these polymers organize into distinct triple helices, single helices, or random coils depending on their primary structure and solvent environment (Figure 2) [20].

The specific physical geometry of the molecule alters its physiological mechanisms and clinical impact. High molecular weight and a triple-helix conformation are critical for receptor cross-linking. For instance, linear structures (cereals) do not trigger the Dectin-1 immune pathway because they lack the specific spatial fit for binding. Also, it generates highly viscous gels in the gastrointestinal tract. This gel layer delays gastric emptying and slows glucose absorption. In the same line, the ratio of tri- to tetra-saccharides (DP3/DP4 units) in the linear chain determines polymer flexibility and water solubility. High molecular weight linear chains optimize entanglement, effectively lowering LDL cholesterol and postprandial glucose spikes. Highly soluble glucans are easily processed for intravenous or oral supplements, whereas insoluble glucans act primarily as dietary fiber or particulate adjuvants. Purely linear glucans (like curdlan) are highly insoluble due to dense inter-chain hydrogen bonding [20].

**Figure 3:**
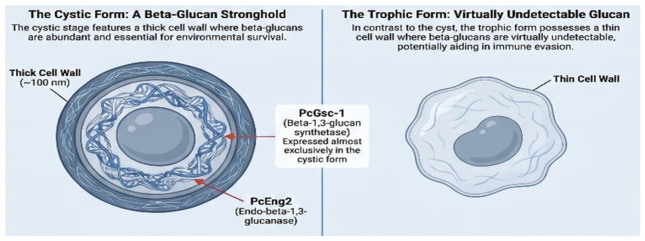
Differential β glucan expression in cystic and trophic forms. The diagram compares the cell wall architecture of the cystic and trophic stages. The cystic form exhibits a thick cell wall (~100 nm) enriched in β glucans, which provide structural resilience and environmental survival. Enzymatic activity is highlighted by PcGsc 1 (β 1,3 glucan synthetase), expressed almost exclusively in the cystic stage, and PcEng2 (endo β 1,3 glucanase). The trophic form, in contrast, displays a thin cell wall with β glucans virtually undetectable, a feature that may contribute to immune evasion. This structural and biochemical dichotomy underscores the role of β glucans in stage specific adaptation and host interaction.

*P. jirovecii* β-glucans have a distinctive molecular architecture with a highly branched network of β-1,3 and β-1,6 bonds, together with a minimal presence of α-glucans and chitin, components that are more common in other environmental fungi and in polysaccharides of dietary origin [2]. This composition undergoes fluctuations throughout the microorganism’s life cycle: cysts, which constitute the infective form, exhibit the highest proportion of these polysaccharides and function as the primary stimulus for immune activation, while trophic forms are nearly devoid of β-glucans (Figure 3) and possess significantly diminished immunogenicity [2,10,11]. This structural and functional differentiation is essential to the recognition process by the innate immune system, particularly through its interaction with receptors such as Dectin-1 and EphA2. The activation of these receptors triggers proinflammatory signaling cascades and cytokine production [3,4].

One study examined the effect of glucan structure on recognition and binding by murine recombinant Dectin-1 with a library of natural product and synthetic (1-->3)-beta/(1-->6)-beta-glucans as well as no glucan polymers. Although Dectin-1 is highly specific for lineal (1-->3)-beta-D-glucans, it does not recognize all glucans equally. Dectin-1 differentially interacted with (1-->3)-beta-D-glucans over a very wide range of binding affinities (2.6 mM–2.2 pM). One of the most striking observations that emerged from this study was the remarkable high-affinity interaction of Dectin-1 with certain glucans (2.2 pM). These data also demonstrated that synthetic glucan ligands interact with Dectin-1 and that binding affinity increased in synthetic glucans containing a single glucose side-chain branch. In the same line, the authors observed differential recognition of glucans derived from saprophytes and pathogens. They found that glucan derived from a saprophytic yeast was recognized with higher affinity than glucan derived from the pathogen *Candida albicans*. Structural analysis demonstrated that glucan backbone chain length and (1-->6)-beta side-chain branching strongly influenced Dectin-1 binding affinity [21].

Dietary β-glucans, derived from cereals, yeasts, or edible fungi, generally have simpler structures, with a reduced degree of branching and variable proportions of β-1,3 and β-1,4 bonds. Moreover, these polysaccharides are frequently linked to other cell wall components, such as α-glucans, proteins, or polysaccharide-protein complexes, which modulate their bioavailability and interaction with immune receptors. Consequently, their immunological recognition is often integrated into immunometabolic regulation circuits, which are predominantly associated with the gut-microbiota axis [4,22].

These structural differences result in markedly different functional profiles. While dietary β-glucans are predominantly associated with immunomodulatory, metabolic, and potentially cardioprotective effects—mediated in part by microbial fermentation, the generation of metabolites with anti-inflammatory activity, and the modulation of innate immunity—β-glucans from *P. jirovecii* configure a signaling pattern characterized by more pronounced inflammatory activation, with the ability to amplify both local and systemic responses [2,3,4,22].

This finding underscores the notion that the biological impact of β-glucans is not solely determined by their chemical composition, but rather by a complex interplay between molecular structure, exposure context, and predominant biological compartment [4,22].

Overall, the high affinity for receptors associated with proinflammatory signaling, the complex β-1,3/β-1,6 branching, and the relative absence of structural components with a buffering effect give *P. jirovecii* β-glucans a particularly efficient profile for inducing amplified immune responses [2]. This phenomenon stands in contrast to the effects of dietary β-glucans, which have been traditionally associated with regulatory and potentially cardioprotective effects that are mediated by microbiota and modulation of innate immunity [4,21]. This functional differentiation provides a framework for understanding fungal β-glucans effects. Their exposure could contribute to systemic inflammatory processes and vascular dysfunction [2,3,4,22].

β-glucans from yeast can induce trained immunity in in vitro and in vivo models, enhancing host defense against pathogens. Despite, intraperitoneal doses of β-glucans in mammals have shown to induce trained immunity, the training effects of orally administering β-glucans are uncommon reported [23,24,25,26]. For example, in turbot, intraperitoneal injection of 50 μL of yeast β-glucan (20 mg/mL) significantly reduced mortality after bacterial infection [23]. Similarly, intraperitoneal administration of 0.1 mg of zymosan (a β-glucan-rich cell wall preparation) into mice markedly increased myeloperoxidase activity and prevented peritonitis for up to 5 weeks [24]. In weaned rabbits, intraperitoneal injection of β-glucan (50 mg/kg) 6 and 4 days before weaning significantly reduced post-weaning diarrhea rates [25]. Finally, newborn goats stimulated with two doses (day −7 and −4) of β-Dh (50 mg/kg) and challenged (day 0) with LPS showed an increase in respiratory burst activity, IL-1β, IL-6, and TNFα production in plasma, and transcription of the macrophage surface markers [26].

### 3.3. Inflammatory Pathways That Are Activated by Pneumocystis jirovecii β-Glucans and Their Possible Contribution to a Proatherogenic Phenotype

*P. jirovecii* β-glucans have been shown to possess a high capacity to activate pattern recognition receptors in macrophages and pulmonary epithelial cells [2,3]. This activation triggers multiple inflammatory pathways, which, although initially oriented towards antifungal defense, can acquire systemic projection and promote processes involved in atherogenesis [2,3,4].

**Figure 4:**
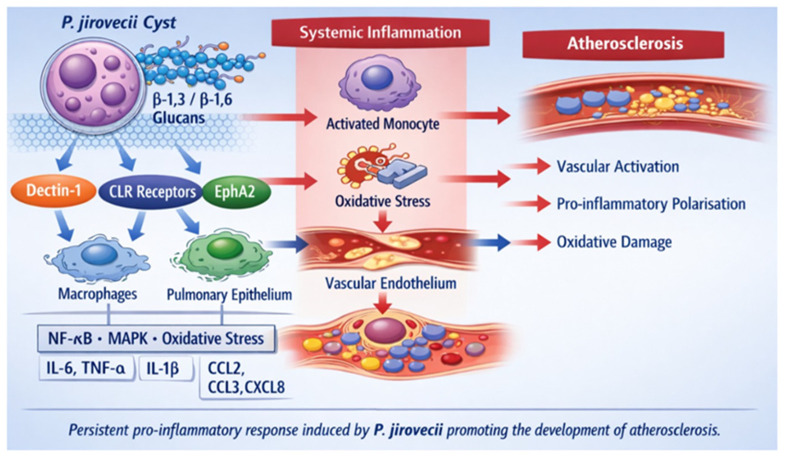
Proposed immunopathogenic model linking *Pneumocystis jirovecii* colonization to systemic inflammation and atherogenesis. The highly branched β-1,3/β-1,6 glucans of the cyst wall engage pattern recognition receptors including Dectin 1, other C type lectin receptors (CLRs), and the tyrosine kinase receptor EphA2. Activation of these pathways triggers intracellular signalling cascades (NF κB, MAPK, and oxidative stress routes), leading to cytokine and chemokine release (IL 6, TNF α, IL 1β, CCL2, CCL3, CXCL8). The resulting inflammatory response might extend beyond the pulmonary compartment, promoting endothelial dysfunction, monocyte activation, and oxidative damage—key processes contributing to the development of atherosclerosis.

Based on the accumulated evidence, we hypothesized a sequence of key events (Figure 4). In macrophages, highly branched β 1,3/β 1,6 glucans of the cyst wall may engage Dectin-1 which constitutes a central axis of recognition. Binding to the extracellular domain of this receptor induces phosphorylation of its ITAM-like motif. This leads to the recruitment of Syk with subsequent activation of the CARD9–BCL10–MALT1 complex. This cascade ultimately leads to the activation of NF-κB and MAPK, which in turn promote the expression of IL-6, TNF-α, IL-1β, and various chemokines, including CCL2, CCL3, and CXCL8. At the same time, other pattern recognition receptors including C type lectin receptors (CLRs), and the tyrosine kinase receptor EphA2 can also be involved. The release of immune mediators both amplify the local inflammatory response and promote monocyte mobilization and their polarization toward proinflammatory profiles, a process closely linked to the early stages of atherosclerotic development [27,28,29]. The resulting inflammatory response extends beyond the pulmonary compartment, promoting endothelial dysfunction, monocyte activation, and oxidative damage—key processes contributing to the development of atherosclerosis [2,3,4,27,28,29].

Moreover, in the lung epithelium, β-glucans have been shown to activate EphA2, a tyrosine kinase receptor involved in the recognition of fungal components. This activation triggers intracellular signaling pathways such as NF-κB [3]. This axis has been demonstrated to promote the production of IL-6, CXCL8, and other mediators that facilitate functional communication between the epithelium and immune cells, thereby contributing to the maintenance of a persistent inflammatory state (Figure 4) [2,3,4,29]. This epithelial-immune circuit is especially relevant in prolonged colonization scenarios, where repeated stimulation can lead to low-grade chronic inflammatory signaling with potential systemic consequences [1,2,3,4,29].

An additional component of this response corresponds to the activation of pathways dependent on lactosylceramide, a glycosphingolipid that acts as a signaling platform in the cell membrane. The interaction of β-glucans with these pathways has been shown to promote the activation of NADPH oxidase and the generation of reactive oxygen species, contributing to the establishment of a state of oxidative stress and the amplification of proinflammatory pathways [2,3,4,27,28,29]. This mechanism, described in fungal recognition models, enhances endothelial dysfunction. It also promotes monocyte activation, two central processes in atherogenic progression [1,2,3,4,27,28,29].

The convergent activation of Dectin-1/CARD9, CLRs, EphA2, and lactosylceramide pathways leads to a sustained inflammatory response. This response is characterized by the production of IL-6, TNF-α, and multiple chemokines, along with other cytokines with modulatory functions, such as IL-10 or IL-23. The participation of these latter cytokines may adjust the intensity and profile of the response rather than simply amplifying it [2,3,4,27,28,29]. These mediators both sustain the antifungal response and might promote a proatherogenic phenotype. The promotion of a proatherogenic phenotype would be achieved through the following mechanisms: endothelial activation, inflammatory recruitment and polarization of monocytes/macrophages, and increased oxidative stress. Consequently, the induction of signaling by β-glucan in the pulmonary compartment might have some effects beyond this organ, thereby contributing to systemic processes associated with vascular inflammation and the development of atherosclerosis [1,2,3,4,27,28,29].

### 3.4. The Effects of β-Glucans on Cardiovascular Risk, with a Particular Focus on the Contrast Between Dietary Effects and Systemic Fungal Exposure

β-glucans have been the subject of extensive research as a potential modulator of cardiovascular risk. Dietary β-glucans predominantly induce regulatory immune interactions rather than intense inflammatory responses [4,19]. Consequently, their cardiometabolic impact is linked to modulation of the gut microbiota and regulation of lipid metabolism. They also attenuate systemic inflammation, mechanisms that converge in an overall cardioprotective profile [13,14,22].

In the gastrointestinal microbiota, dietary β-glucans act as fermentable substrates. They promote the growth of short-chain fatty acid-producing bacteria, including *Faecalibacterium*, *Roseburia*, and *Bifidobacterium*. These metabolites, particularly butyrate and propionate, exert anti-inflammatory effects. They reinforce intestinal barrier integrity and modulate metabolic pathways involved in lipid and carbohydrate homeostasis [4,20]. These changes are associated with reductions in LDL-C and triglycerides, improved insulin sensitivity, and decreased circulating inflammatory markers, as well as slower progression of atherosclerotic lesions in experimental models [14,22].

Conversely, circulating fungal β-glucans have high affinity for proinflammatory receptors. These include Dectin-1, CLRs, and EphA2 thereby facilitating their recognition by the innate immune system [2,3,4]. Upon entering the circulation, these polysaccharides activate the Syk–CARD9–NF-κB axis, thereby promoting the production of proinflammatory cytokines, such as IL-6, TNF-α, and IL-1β, as well as various chemokines, and stimulating the activation of circulating monocytes [27,28,29]. This response, initially directed toward antifungal defense, may persist systemically following antigen exposure during chronic infection, thereby promoting a proatherogenic phenotype characterized by sustained inflammation and vascular activation [2,3,4,14,27,28,29].

Furthermore, fungal β-glucans contribute to endothelial dysfunction by activating lactosylceramide-dependent pathways. This leads to reactive oxygen species generation which, in turn, has been demonstrated to promote the expression of endothelial adhesion molecules, monocyte recruitment, and the progression of atherosclerotic lesions [2,3,4,27,28,29]. In contrast to dietary β-glucans, which modulate the inflammatory response into a regulatory profile, circulating fungal β-glucans have been observed to amplify systemic inflammation and oxidative stress. These phenomena are two processes closely linked to vascular vulnerability [1,2,3,4,27,28,29].

From an integrative perspective, intestinal exposure to dietary β-glucans has been associated with protective metabolic and anti-inflammatory signals; however, systemic exposure to fungal β-glucans in invasive infections or persistent colonization scenarios may promote low-grade chronic inflammation, endothelial dysfunction, and a molecular environment conducive to atherogenesis [2,3,4,14,22,29]. This distinction is especially important when interpreting inflammatory biomarkers and exploring emerging mechanisms of vascular inflammation associated with subclinical infections [2,3,4,14,29].

### 3.5. The Serum Persistence of (1→3)-β-D-Glucan in Pneumocystis jirovecii, Encompassing Its Progression from Chronic Colonization to Systemic Inflammation and Vascular Dysfunction

Serum (1→3)-β-D-glucan is a widely used biomarker for diagnosing invasive fungal infections, including *P. jirovecii* pneumonia. However, it is not found in all fungal species and cannot be used to identify infections by *Cryptococcus*, *Blastomyces* (yeast form), or *Zygomycetes* such as *Absidia*, *Mucor*, or *Rhizopus* since these genera either do not produce β-D-glucan or produce low levels that might lead to false negatives [30].

The primary clinical diagnostic limitation of β-D-glucan testing is its high susceptibility to both analytical and biological noise. Because the test operates as a functional kinetic bioassay—traditionally relying on the Limulus amebocyte lysate cascade triggered by the G factor—it frequently identifies β-D-glucan from non-fungal origins. This diagnostic noise leads to elevated false-positive rates, which complicates clinical decision-making and limits the test’s positive predictive value [30].

β-D-glucan is a structural component of cellulose. Transient, false-positive elevations frequently occur from standard medical supplies like sterile gauze, cotton swabs, hemodialysis cellulose membranes, and non-β-D-glucan-free collection tubes. In addition, patients undergoing chemotherapy, or those with severe mucosal barrier injury (e.g., mucositis, gut ischemia), can experience microbiome-derived β-D-glucan translocation into the bloodstream. This triggers a positive result in the absolute absence of a systemic fungal infection. In addition, certain bacterial infections—specifically hypermucoid strains like *Pseudomonas aeruginosa* or *Streptococcus pneumoniae*—produce linear or cyclic glucans that trigger the assay. Finally, intravenous amoxicillin-clavulanate, piperacillin-tazobactam, and human blood products (fractionated albumin or immunoglobulins) similarly feed into the background noise [30].

Clinicians should also be cautious of scenarios that may lead to false-positive results. Awareness of the factors that can contribute to such non-Invasive Fungal Disease (IFD)-related findings can enhance the planning and interpretation of β-D-glucan assays and support investigational strategies, such as serial sampling and β-D-glucan clearance evaluation, to assess the likelihood of contamination and improve patient care.

However, beyond its diagnostic value, the magnitude and, in particular, the persistence of its positivity could reflect a state of immunologically active exposure to fungal β-glucans with possible systemic consequences. This consideration is especially important in scenarios of chronic or subclinical colonization by *P. jirovecii*, in which the absence of overt clinical manifestations does not preclude the existence of persistent low-grade inflammatory stimulation [2,6,28].

Montes Cano et al. demonstrated that colonization by *P. jirovecii* can exhibit dynamic patterns. These include cycles of acquisition, loss, and accelerated recolonization by different genotypes, even in individuals without overt clinical disease [26]. These cycles may cause fluctuations in β-glucan release into the pulmonary compartment. This can result in persistent or intermittent serum positivity. This, in fact, reinforces the hypothesis of sustained antigenic exposure with the capacity to modulate systemic inflammation [2,6,28,31,32].

Several studies have documented that patients with *P. jirovecii* pneumonia, as well as colonized individuals—particularly the elderly, individuals with chronic obstructive pulmonary disease, pulmonary fibrosis, or mild immunosuppression—may have serum concentrations of (1→3)-β-D-glucan that are elevated and sustained even after clinical resolution of the respiratory episode [2,6]. This finding suggests that β-glucans may translocate from the pulmonary compartment to the systemic circulation. This process could be sustained over time, either due to persistence of the microorganism, slow replacement of cell wall components, or alterations in the alveolar–capillary barrier associated with chronic inflammation [2,6,28,33].

Pathophysiologically, circulating β-glucans are not merely passive markers of exposure, but rather active immunological stimuli capable of interacting with pattern recognition receptors expressed on immune and vascular cells, including Dectin-1, various CLRs, and EphA2 [2,3,4,27,28,29]. Sustained activation of these signaling axes can drive a chronic proinflammatory state, characterized by the production of cytokines such as IL-6, TNF-α, and IL-1β, as well as the release of chemokines that promote the recruitment and activation of circulating monocytes [2,3,4,27,28,29,30,31,32,33].

This state of low-grade systemic inflammation is highly relevant to vascular biology. Chronic exposure of the endothelium to inflammatory mediators derived from β-glucan-induced activation may be associated with endothelial dysfunction, increased expression of adhesion molecules such as VCAM-1, ICAM-1, and E-selectin, and alterations in the bioavailability of nitric oxide [2,14,27]. These alterations promote the adhesion and transmigration of monocytes to the arterial intima, a pivotal event in the initial phases of atherogenesis [2,14,28,33].

It has been proposed that circulating β-glucans may influence platelet activation and coagulation, either directly or indirectly through systemic inflammation and oxidative stress. In this context, the activation of the endothelium and myeloid cells may promote a procoagulant environment, characterized, among other changes, by an increase in tissue factor expression [2,14,28,33].

### 3.6. Experimental Models to Explore a Potential Causal Link Between Pneumocystis jirovecii β-Glucans and Atherosclerosis

Evidence from animal studies provides strong mechanistic support for a causal link between *Pneumocystis jirovecii* and atherogenesis. Early work demonstrated that *Pneumocystis* cell wall β glucans can stimulate alveolar macrophages to release arachidonic acid and its metabolites, initiating inflammatory cascades relevant to vascular injury [34]. Subsequent murine and in vitro studies confirmed that β glucans induce dendritic cell activation and costimulatory molecule expression, thereby amplifying systemic inflammation [35]. These findings highlight a biologically plausible mechanism by which chronic fungal exposure could accelerate endothelial dysfunction and plaque formation.

In humans, the evidence is more indirect. β glucans from *Pneumocystis* have been shown to stimulate calcium-dependent signaling and IL 8 secretion in airway epithelial cells, linking colonization to persistent inflammatory responses [36]. While serum β glucanemia has been associated with systemic low-grade inflammation and endothelial activation, no prospective studies have yet demonstrated a direct progression from *P. jirovecii* colonization to cardiovascular events. Thus, while the human data support a biologically plausible association, they remain insufficient to establish causality.

Taken together, results obtained in animal models provide strong mechanistic evidence for a causal relationship, whereas human studies highlight associations consistent with atherogenic pathways but not yet definitive. This dual perspective underscores the need for translational and longitudinal research to clarify whether *P. jirovecii* colonization contributes significantly to cardiovascular disease in humans. The establishment of a causal relationship between systemic exposure to *P. jirovecii* β-glucans and the progression of atherosclerosis necessitates the development of experimental models that coherently integrate immunological, vascular, and metabolic components. Given that *P. jirovecii* is a strictly human fungus that cannot be cultured by conventional methods, available approaches must focus on the controlled administration of purified β-glucans or on models that reproduce their key immunobiological effects [2,3,4,27,28,29,30,31,32,33,34,35,36,37]. A “humanized mouse” (mouse with human immune system) might be a future direction to bridge the gap between *P. murina* models and human pathology.

Nevertheless, this constraint does not preclude the utilization of complementary methodologies. The utilization of models employing *Pneumocystis carinii* in rats and *P. murina* in mice, which are extensively employed in preclinical research, facilitates the analysis of responses elicited by the entire microorganism. These models provide a comparative framework for examining the contribution of β-glucans to pulmonary and systemic inflammation. In this context, several reviews emphasize that β-glucans, as abundant components of the *Pneumocystis* cell wall, interact with multiple receptors of the innate immune system and activate proinflammatory signaling networks that cannot be reproduced using axenic cultures of *P. jirovecii* [2,3,4,27,28,29,30,31,32,33]. Conversely, the integration of animal models with elevated physiological intricacy, such as porcine models of atherosclerosis, has the potential to enable the assessment of vascular and metabolic ramifications within a cardiovascular system that more closely resembles that of humans. This development offers a way for the exploration of the association between exposure to fungal β-glucans and atherosclerotic progression with enhanced precision [31,32,33,34,35,36,37].

### 3.7. Murine Models of Atherosclerosis

Genetically modified murine models, particularly ApoE^−/−^ and LDLR^−/−^ mice, have been extensively validated as platforms for studying atherogenesis and the interaction between systemic inflammation and vascular biology [37]. In this context, the repeated administration of *P. jirovecii* β-glucans—either via intratracheal or by parenteral routes—would facilitate the evaluation of their impact on the burden and composition of atherosclerotic plaques, immune cell infiltration, and the expression of inflammatory and oxidative stress markers in the arterial wall [2,21,22,23,24,25,26,27,28,29,30,31,32,33,34,35,36,37,38,39].

Similarly, in studies to assess exposure to the complete microorganism, infection models with *P. murina* or *P. carinii* can be utilized. These models facilitate complementary exploration of the contribution of the *Pneumocystis*-induced inflammatory response to vascular dysfunction and the progression of atherosclerosis, integrating both β-glucan-mediated signaling and the participation of other components of the fungal wall [2,3,4,23,24,25,26,27,28,29,30,31,32,33,34,35,36,37,39].

In this regard, robust activation of the NLRP3 inflammasome and NETosis-associated pathways has been described in mice infected with *P. murina*, with the generation of intense pulmonary inflammatory responses and local microvascular alterations [30]. In accordance with this observation, murine models of chronic respiratory diseases have demonstrated that *Pneumocystis* infection exacerbates perivascular inflammation and immune cell infiltration around the vessels. This finding suggests a possible convergence with mechanisms involved in endothelial dysfunction and chronic vascular disease progression [31,32,33,39,40].

### 3.8. Cell Cultures and In Vitro Vascular Models

In vitro models provide a complementary approach to elucidating mechanisms at higher molecular resolution. Human endothelial cell cultures exposed to *P. jirovecii* β-glucans allow the evaluation of changes in endothelial activation, reactive oxygen species production, adhesion molecule expression, and alterations in barrier function [2,3,4,27,28,29,33,39]. The integration of co-culture systems with monocytes or macrophages facilitates the analysis of cellular interactions pertinent to the initial phases of atherosclerotic injury. This approach integrates CLR- and CARD9-mediated signaling pathways, as well as additional inflammatory mechanisms previously described in *Pneumocystis* infection models. These mechanisms include NLRP3 inflammasome activation and NETosis processes, which have the potential to amplify oxidative stress and endothelial dysfunction [39,40,41].

Conversely, the utilization of three-dimensional endothelial models or microfluidic systems that replicate laminar flow and shear stress conditions could yield more physiologically accurate data concerning the way exposure to β-glucans influences the endothelial response within an environment analogous to the in vivo vascular environment [2,3,4,27,28,29,33,39].

### 3.9. Integration with Immunometabolic and Omic Profiles

A contemporary experimental approach necessitates the integration of the analysis of transcriptomic, epigenetic, and metabolic profiles in response to exposure to *P. jirovecii* β-glucans, ideally combined with integrative analysis and computational modeling strategies. The characterization of the plasticity of monocytes and macrophages towards proinflammatory or proatherogenic phenotypes, as well as the identification of molecular signatures associated with vascular inflammation, could reinforce the biological plausibility of the proposed link and facilitate the construction of predictive models that capture complex interactions between immunometabolic pathways and disease progression. In this context, the combination of animal models, omic analyses, and measurement of circulating biomarkers—including cytokines, chemokines, and markers of endothelial activation—would allow parallels to be drawn with clinical observations and advance toward a more robust translational integration [27,33,37,40].

### 3.10. Methodological Limitations and Considerations

Recent evidence in mouse models demonstrates that animals pre-treated with dietary β-glucans exhibit significantly higher survival rates and a lower bacterial load when exposed to infections caused by *Staphylococcus aureus*, *Candida albicans* or parasites. Furthermore, it has been observed that they enhance the recruitment of NK (Natural Killer) cells and promote the apoptosis of neoplastic cells in experimental cancer models [2].

It is important to note that microorganisms in the caecum and colon ferment dietary β-glucans, exponentially increasing the concentration of short-chain fatty acids (SCFAs) such as acetate, propionate and butyrate. The increase in SCFAs optimizes the integrity of the intestinal barrier by increasing the expression of tight junction proteins such as occludin, preventing bacterial translocation and reducing inflammation. However, few studies on the impact of dietary β-glucans in animal models have been reported [2].

Despite taxonomic barriers, rodent models are the gold standard for studying *Pneumocystis* pneumonia. The broad immunological mechanisms—such as the requirement of CD4+ T cells to clear infection—are functionally analogous. The murine model (*P. murina*) is extensively used to study host–pathogen interactions and immune reconstitution [2,3,11,41]. Also, studies from rodent-specific *Pneumocystis* species (like *Pneumocystis carinii* in rats or *P. murina* in mice) to humans—who are exclusively infected by *Pneumocystis jirovecii*—is a common but highly nuanced practice. While rodent models remain indispensable for immunological and pre-clinical research, strict biological differences limit direct clinical translation [2,3,11,41].

Notwithstanding their value, these models are subject to inherent limitations. The extrapolation of findings from murine models to human subjects necessitates caution, particularly within the context of antifungal immunity. Furthermore, the structural heterogeneity of β-glucans, in conjunction with variations in dosage, route of administration, and duration of exposure, can exert a substantial influence on experimental outcomes. In this regard, the standardization of *P. jirovecii* β-glucan preparations and the detailed characterization of their structure emerge as critical requirements for the reproducibility and proper interpretation of studies [2,33,39,40].

A main concern is the bias to extend mechanistic plausibility toward implicit clinical applicability. While experimental studies demonstrate that β-glucans activate inflammatory pathways implicated in atherogenesis, this evidence is largely derived from in vitro and animal models. There is currently no clinical or epidemiological evidence showing that *P. jirovecii* colonization or β-glucan exposure influences the development or progression of atherosclerotic disease in humans.

### 3.11. Experimental Synthesis and Translational Projection

The use of murine models of atherosclerosis, vascular cell systems, and integrated omics approaches is a coordinated effort that provides a robust experimental framework for testing the hypothesis that *P. jirovecii* β-glucans act not only as markers of fungal exposure but also as active modulators of vascular inflammation and atherosclerotic progression. Overall, the results might provide crucial advancements from observational associations and biological plausibility toward a more causal comprehension of the intersection between antifungal immunity and cardiovascular risk [26,33,39,40].

This approach is particularly relevant in populations with persistent or repeated exposure to *P. jirovecii*, such as older adults and patients with chronic obstructive pulmonary disease, pulmonary fibrosis, or mild immunosuppression, in whom subclinical colonization and low-grade inflammation could acquire greater systemic relevance [32,33,39,40,41].

### 3.12. The Pathophysiological and Clinical Implications of Pneumocystis jirovecii β-Glucans in Atherogenic Inflammation

An integrative review of the extant literature was conducted, and the evidence supports a pathophysiological model in which *P. jirovecii* β-glucans function not only as markers of fungal exposure but also as active mediators of systemic inflammation with potential vascular repercussions. In contrast to dietary β-glucans, whose immunometabolic effects are frequently linked to modulatory and potentially cardioprotective profiles, *P. jirovecii* β-glucans possess structural characteristics that promote sustained activation of proinflammatory pathways, particularly in scenarios of persistent colonization or active infection [2,33,39,40].

From a pathophysiological perspective, the activation of receptors such as Dectin-1, other C-type lectins (CLRs), and EphA2 constitutes a central axis in the transduction of signals induced by highly branched β-glucans. The interplay between Syk-CARD9-NF-κB-dependent signaling and the activation of MAPK, STAT3, and oxidative stress-related pathways has been demonstrated to promote the production of key cytokines and chemokines, including IL-6, TNF-α, IL-1β, and CXC chemokine ligand 8 (CXCL8). This process has been shown to modulate both pulmonary innate immunity and the systemic inflammatory response. The prolonged presence of these signals has the potential to induce alterations in the plasticity of monocytes and macrophages, leading to a proinflammatory phenotype. This process is intricately associated with the progression of atherosclerotic lesions [2,33,39,40].

In this context, the lung emerges as a key immune node from which inflammatory signals can be amplified and disseminated systemically. In vulnerable populations, such as older adults, individuals with chronic lung disease, or those with mild immunosuppression, subclinical colonization by *P. jirovecii* has the potential to generate chronic low-grade inflammatory stimulation. This stimulation can be sufficient to sustain systemic inflammation, oxidative stress, and endothelial activation in the absence of obvious infectious manifestations [32,33,40,41]. This scenario is particularly salient in the context of atherosclerosis, a chronic inflammatory disease in which persistent endothelial activation, monocyte recruitment, and vascular dysfunction play a pivotal role [1,32,33,41].

Within the interpretative framework outlined, serum positivity for (1→3)-β-D-glucan assumes a more intricate dimension. Beyond its diagnostic utility in invasive fungal infections, the magnitude and duration of its elevation could reflect immunologically relevant exposure to circulating β-glucans, with the capacity to modulate vascular biology [5,33]. The potential correlation between prolonged levels of β-D-glucan, endothelial activation, coagulation alterations, and atherosclerotic plaque progression gives rise to clinical inquiries that exceed the scope of infectious diseases and extend into the domain of cardiovascular medicine [5,33,39,41].

The comparison with dietary β-glucans underscores the significance of origin and structural properties in determining biological effects. While β-glucans from cereals and edible fungi have a weaker interaction with the immune system and exert beneficial effects mediated, in part, by the gut microbiota and metabolic modulation, β-glucans from *P. jirovecii* lack structural elements that limit their immune recognition and show a high affinity for proinflammatory receptors. This functional divergence underscores the need to avoid simplistic interpretations that equate all β-glucans as biologically equivalent entities [2,33,39,41].

From an experimental perspective, murine models of atherosclerosis, in vitro endothelial systems, and integrated omics approaches offer tools to move from observational associations to pathophysiological inferences. The ability to analyze specific pathways, such as those dependent on Dectin-1/CARD9 or lactosylceramide, will facilitate the identification of critical signaling nodes susceptible to intervention and elucidate the extent to which exposure to fungal β-glucans directly contributes to vascular inflammation [26,33,39,41].

However, it must be acknowledged that this review is subject to inherent limitations, primarily due to its narrative nature and the heterogeneity of the available studies. A significant proportion of the extant evidence derives from experimental models or observational studies with limited sample sizes; consequently, extrapolation to human populations should be done with caution. Additionally, the standardized quantification of *P. jirovecii* β-glucans and the distinction between transient and persistent exposure remain significant methodological challenges [2,33,39,41].

To date, clinical evidence does not allow for definitive conclusions regarding a potential causal relationship between exposure to *P. jirovecii* β-glucans and the occurrence of cardiovascular events. However, the available experimental models provide a sufficient framework for the controlled evaluation of this hypothesis [33,39,40,41].

Concurrently, numerous studies have documented a high prevalence of *P. jirovecii* colonization in various populations, including individuals with HIV, patients with COPD, infants, and notably, the general population. The near ubiquity of the detection of *Pneumocystis* in the lungs of infants who died of sudden death syndrome suggests that subclinical exposure is a widespread phenomenon and potentially relevant from a pathophysiological point of view [32,33,41,42].

*Pneumocystis* colonization acts as a highly reactive catalyst when combined with smoking or diabetes. Smoking damages mucosal cilia and hinders lung clearance; this creates an ideal environment for *Pneumocystis* to colonize and persist [42]. Hyperglycemia impairs basic immune functions, compromising T-lymphocyte and natural killer (NK) cell activity. This allows *Pneumocystis* to maintain a higher subclinical fungal burden. Diabetes also produces advanced glycation end-products (AGEs), which cause tissue stiffness and inflammation. The combination of AGE-induced stress and *Pneumocystis*-induced IL-1 activation creates a severe, dual-source inflammatory environment that accelerates vascular and systemic complications [43].

In the same line, *Pneumocystis* colonization directly accelerates and worsens the classic lipid-driven vascular damage pathway. It moves the needle from “stable” dyslipidemia to aggressive tissue damage through several specific mechanisms. In dyslipidemia, macrophages process oxidized LDL (oxLDL) via scavenger receptors (like CD36), eventually transforming into plaque-forming foam cells. In this context, constant fungal engagement triggers the downstream release of IL-1β, TNF-α, and monocyte chemotactic protein-1 (MCP-1). Local and circulating MCP-1 recruits more monocytes to the vascular intima, while TNF- α upregulates scavenger receptors on those cells. This causes them to engulf oxLDL at an accelerated rate, dramatically speeding up foam cell formation [44].

*Pneumocystis* cell wall components stimulate macrophages and neutrophils to produce high amounts of reactive oxygen species (ROS). When this heavy local ROS production spills into systemic circulation, it directly oxidizes native circulating LDL into its highly atherogenic, cytotoxic oxLDL form, worsening existing dyslipidemia [44].

Beyond fueling the lipid pathway, *Pneumocystis* drives separate, parallel cellular damage mechanisms that compound cardiovascular and tissue risk. *Pneumocystis* colonization characteristically shifts the local immune environment toward a chronic Th17 and Th1 response, marked by high levels of IL-17 and IL-23 [2]. This specific cytokine profile functions independently of cholesterol. It acts directly on the vascular endothelium, increasing the expression of VCAM-1 and prompting smooth muscle cell proliferation—the structural root of arterial wall thickening [33].

The coexistence of frequent fungal colonization and atherosclerotic disease of high prevalence does not, in itself, imply a causal relationship. However, it does establish a biologically plausible scenario that justifies systematic investigation. In this regard, prospective studies assessing the presence of *P. jirovecii* or its immunoactive components in patients with atherosclerosis, together with longitudinal monitoring of colonization, would constitute a reasonable methodological approach to explore this potential immunobiological interaction [31,33,41,42].

In fact, with advances in antiretroviral therapy, most deaths in people with HIV are now attributable to noncommunicable illnesses, especially cardiovascular disease. A recent review examined the epidemiology and clinical features of cardiovascular disease, with particular emphasis on coronary heart disease in the context of HIV infection, highlighting a substantially increased risk of myocardial infarction even when HIV infection is well controlled [45]. In this context, *P. jirovecii* remains one of the most important pathogens in people living with HIV [5]. Based on the hypothesis presented in this document, it would be worthwhile to conduct studies on atherosclerosis in people living with HIV in whom colonization by *P. jirovecii* is significant.

## 4. Perspectives

Current evidence suggests that *Pneumocystis jirovecii* β-glucans may be an underrecognized contributor within the inflammatory continuum linking pulmonary immunity to vascular pathology. Yet, their clinical significance remains insufficiently defined. Several lines of investigation emerge as essential to advance this field.

A primary priority is the distinction between transient and persistent exposure. The sustained presence of circulating (1→3)-β D glucan, even in the absence of overt clinical manifestations, raises the possibility of chronic immunological stimulation with systemic repercussions [5,33]. Developing standardized methods capable of quantifying *P. jirovecii* specific β glucans—and differentiating them from other fungal sources—will be crucial for establishing causal inferences [33,39,41]. Recent studies have shown that novel mouse monoclonal antibodies (such as mAbs 3G11 and 5H5) selectively bind to the branched structure of fungal glucans while completely ignoring the linear glucans produced by bacteria (e.g., *Pseudomonas aeruginosa* and *Alcaligenes faecalis*) [46]. Incorporating these mAbs into enzyme-linked immunosorbent assays or lateral flow formats can shield the diagnostic process from iatrogenic or bacterial cross-reactivity. Future clinical trial frameworks should evaluate a dual-triage diagnostic pathway. In this model, the inexpensive, automated β D glucan assay serves as an initial rule-out mechanism, with any positive results automatically triggering a reflex confirmation test using highly specific monoclonal antibody-targeted PCR panels. This dual approach mitigates the clinical noise of false positives and prevents the unnecessary use of empiric antifungals.

Equally important is the refinement of mechanistic understanding. Highly branched β glucans from *P. jirovecii* activate a network of proinflammatory pathways, including Dectin 1/CARD9-dependent signaling, MAPK and STAT3 activation, and oxidative stress-related cascades [33,39,40]. Dissecting these circuits through murine models of atherosclerosis, endothelial cell systems, and integrated omics approaches will help identify critical signaling nodes amenable to therapeutic modulation and clarify whether fungal β glucans directly contribute to vascular inflammation [28,33,39,40].

From an epidemiological standpoint, the high prevalence of subclinical colonization in vulnerable populations—older adults, individuals with chronic lung disease, those with mild immunosuppression, and people living with HIV—raises clinically relevant questions [32,33,37,42]. In these groups, the coexistence of frequent colonization and elevated cardiovascular risk creates a biologically plausible scenario that warrants prospective evaluation. Notably, in people living with HIV, where cardiovascular disease has become a leading cause of morbidity and mortality despite virological control, the potential contribution of *P. jirovecii* colonization deserves systematic investigation [5,43].

The comparison with dietary β glucans further underscores the need to abandon generalized assumptions of biological equivalence. Structural features that determine receptor affinity and immunological effect differ markedly between fungal and dietary β glucans, and elucidating these distinctions may open new avenues for selective immunomodulation [33,39,40].

Collectively, these perspectives outline an emerging research landscape at the intersection of pulmonary immunology, vascular biology, and medical mycology. Clarifying the role of *P. jirovecii* β glucans in systemic inflammation and cardiovascular risk has the potential not only to refine current models of atherogenesis but also to reveal novel diagnostic and therapeutic opportunities for high-risk populations.

## 5. Conclusions

The β-glucans of *P. jirovecii* may be conceptualized as active mediators of systemic inflammation with potential relevance for vascular biology and atherogenesis. Their highly branched architecture—dominated by β-1,3/β-1,6 motifs—and their interaction with proinflammatory receptors such as Dectin-1, various CLRs, and EphA2 promote sustained activation of monocytes, macrophages, and endothelial cells. This fosters an immunoinflammatory environment with pro-atherogenic characteristics, in contrast to the modulatory profiles described for dietary β-glucans.

The persistence of (1→3)-β-D-glucan in serum suggests subclinical or chronic exposure to fungal polysaccharides, potentially associated with low-grade inflammation, oxidative stress, platelet activation, and endothelial dysfunction—processes that converge in the progression of atherosclerotic lesions. Although most of the available evidence derives from experimental models and observational studies, the body of data establishes a coherent conceptual framework linking pulmonary antifungal immunity with mechanisms of systemic vascular inflammation.

While the accumulated evidence underscores the biological plausibility of *P. jirovecii* β-glucans contributing to systemic inflammation and vascular dysfunction, the strength of this association varies considerably across levels of evidence. Experimental studies provide robust mechanistic insights, demonstrating receptor engagement and downstream inflammatory cascades, yet these findings are largely confined to in vitro systems and rodent models. Observational data highlight colonization and biomarker persistence, but remain indirect and heterogeneous, with limited capacity to establish causality. Importantly, the absence of prospective human studies linking *P. jirovecii* exposure to cardiovascular outcomes underscores a critical gap. Thus, current knowledge should be interpreted as a framework of biologically plausible mechanisms rather than definitive clinical evidence, reinforcing the need for translational research that bridges experimental immunology with epidemiological validation (Table 1).

In this context, studies that discriminate between transient and persistent exposure, validate biomarkers, and explore specific pathophysiological pathways will be essential to more precisely delineate the clinical impact of *P. jirovecii* β-glucans on cardiovascular risk. Finally, the development of new humanized monoclonal antibodies, as well as novel and standardized PCR methods that can quantify *P. jirovecii*-specific β-glucans and distinguish them from other nonfungal and dietary sources, is an essential tool for establishing a causal inference.

## Figures and Tables

**Table 1:** Evidence framework on *P. jirovecii* β-glucans and cardiovascular risk.

Category	Key Findings	Representative Evidence
**Experimental findings**	*P. jirovecii* β-glucans activate Dectin-1, EphA2, CLRs, and lactosylceramide pathways in macrophages and lung epithelium. Induction of NF-κB, MAPK, STAT3 cascades → secretion of IL-6, TNF-α, IL-1β, CCL2, CXCL8. Promote oxidative stress via NADPH oxidase and ROS generation. Experimental models show link between fungal β-glucans and atherosclerosis.	Preclinical studies in macrophages, epithelial cells, and animal models [2,3,4,27,28,29].
**Observational evidence**	Colonization by *P. jirovecii* is frequent in chronic respiratory diseases (COPD, cancer, HIV). Serum (1→3)-β-D-glucan persists even without overt infection, indicating systemic exposure. Colonization associated with low-grade inflammation and altered immune microenvironment.	Clinical observations and biomarker studies [5,6,7,19].
**Hypothesis-generating concepts**	Persistent exposure to cyst-derived β-glucans may amplify pulmonary inflammation and extend systemically. This chronic low-grade inflammation could contribute to endothelial dysfunction and monocyte/macrophage polarization. Dietary β-glucans contrast sharply, showing immunoregulatory and cardioprotective effects via microbiota and metabolism. Distinguishing fungal vs. dietary β-glucans is critical for interpreting vascular inflammation mechanisms.	Conceptual synthesis from narrative review; biologically plausible but not yet epidemiologically proven [1,2,3,4,12,13,14,22].

## Data Availability

Not applicable.

## Abbreviations

The following abbreviations are used in this manuscript:

Receptors and Recognition Molecules	
CLRs	C type lectin receptors
Dectin 1	C type lectin receptor Dectin 1
EphA2	Ephrin type A receptor 2
TLR2/TLR4	Toll like receptors 2 and 4
Signaling Pathways and Intracellular Complexes	
CARD9	Caspase recruitment domain containing protein 9
BCL10	B cell lymphoma/leukemia 10
MALT1	Mucosa associated lymphoid tissue lymphoma translocation protein 1
ITAM like	Immunoreceptor tyrosine-based activation motif like
Syk	Spleen tyrosine kinase
MAPK	Mitogen activated protein kinase
NF κB	Nuclear factor kappa light chain enhancer of activated B cells
STAT3	Signal transducer and activator of transcription 3
NLRP3	NOD like receptor family pyrin domain containing 3
Cytokines, Chemokines, and Inflammatory Mediators	
IL 1β	Interleukin-1 Beta
IL 6	Interleukin 6
TNF α	Tumor Necrosis Factor Alpha
CXCL8	C X C motif chemokine ligand 8
CCL2/CCL3	C C motif chemokine ligands 2 and 3
IL 10	Interleukin 10
IL 23	Interleukin 23
MCP-1	Monocyte chemotactic protein-1
Endothelial Adhesion Molecules	
VCAM 1	Vascular cell adhesion molecule 1
ICAM 1	Intercellular adhesion molecule 1
Animal Models and Experimental Genetics	
ApoE^−^/^−^	Apolipoprotein E knockout mouse
LDLR^−^/^−^	Low density lipoprotein receptor knockout mouse
Other abbreviations	
AGEs	Glycation end-products
ROS	Reactive oxygen species
oxLDL	Oxidized LDL
PCP	*Pneumocystis* pneumonia
